# Methylphenidate Dose-Dependently Affects Aggression and Improves Fear Extinction and Anxiety in BALB/cJ Mice

**DOI:** 10.3389/fpsyt.2019.00768

**Published:** 2019-10-25

**Authors:** Amanda Jager, Doranda Kanters, Femke Geers, Jan K. Buitelaar, Tamas Kozicz, Jeffrey C. Glennon

**Affiliations:** ^1^Department of Cognitive Neuroscience, Donders Institute for Brain, Cognition and Behavior, Radboudumc, Nijmegen, Netherlands; ^2^Department of Anatomy, Donders Institute for Brain, Cognition and Behavior, Radboudumc, Nijmegen, Netherlands; ^3^Department of Clinical Genomics, Center for Individualized Medicine, Mayo Clinic, Rochester, MN, United States

**Keywords:** aggression, animal, anxiety, attention-deficit hyperactivity disorder, conduct disorder, methylphenidate

## Abstract

Overt aggression, increased anxiety, and dysfunctional fear processing are often observed in individuals with conduct disorder (CD) and attention-deficit hyperactivity disorder (ADHD). Methylphenidate (MPH), a psychostimulant increasing dopamine and noradrenaline tone, is effective in reducing aggression in both CD and ADHD individuals. However, it is unclear to which extent these effects of MPH are dose dependent. Here, the effects of acute intraperitoneal MPH (3 and 10 mg/kg) on aggression, anxiety, social behavior, and fear extinction were investigated in BALB/cJ mice. Previous studies in BALB/cJ mice have revealed high levels of aggression and anxiety that are associated with reduced top-down cortical control. Administration of 3 mg/kg MPH prolonged the attack latency and prevented escalation of aggression over time compared to vehicle-treated mice, while 10 mg/kg MPH increased number of bites and attacks. In addition, 3 mg/kg MPH decreased social interaction slightly. A strong anxiolytic effect was found after administration of both the 3 and 10 mg/kg doses in the elevated plus maze and the open-field test. In addition, while vehicle-treated BALB/cJ animals showed intact freezing, both doses of MPH decreased freezing to the unconditioned stimulus in a fear-conditioning paradigm. A long-lasting effect on fear extinction was visible after treatment with the 10 mg/kg dose. The data support a role for MPH in the regulation of anxiety, fear processing, and aggression in BALB/cJ mice, with the latter effect in a dose-dependent manner. The findings provide a further context for examining the effects of MPH in clinical disorders such as ADHD and CD.

## Introduction

Children and adolescents showing aggressive and antisocial behavior are an increasing socioeconomic and societal problem, mainly due to the persistent and repeating nature of offences. In particular, heightened levels of aggression are found in children diagnosed with conduct disorder (CD), whether or not in combination with attention-deficit hyperactivity disorder (ADHD) (1). In addition to aggression, these juveniles suffer often from attention deficits, hyperactivity, impulsivity, increased anxiety, and abnormal fear processing (2–5). Finding the right pharmacotherapy remains challenging (6–9).

Current ADHD treatment guidelines recommend the use of methylphenidate (MPH) as the first-line pharmacological treatment (10, 11). Reviews and meta-analyses indicated that MPH has moderate to large effects (effect size between 0.69 and 0.9) on aggression in ADHD and CD patients (12, 13). Conduct symptoms and aggression observed in young CD patients with co-occurring ADHD significantly improve after treatment with MPH, independently of the severity of ADHD symptoms (14). Increased aggression can however also be an adverse effect of psychostimulant treatment, and it is unclear whether this is dose dependent (15). Furthermore, studies addressing the effect of MPH on anxiety are mixed and may depend on the state anxiety of the individual (16–19). In both human and animal studies, the dose of MPH appeared to be related to the behavioral outcome. Inverted U-shaped effects were seen after stimulant treatment in ADHD children, that is, the low dose improved memory tests, whereas the high dose significantly decreased the performance on the task (20). In mice, similarly, a low dose of MPH enhanced fear memory, whereas a high dose impaired memory (21). Other studies have shown a positive dose–response pattern on attention and hyperactivity in children with ADHD (22). However, this was only in a subgroup of ADHD patients, as it is reported that children with ADHD without hyperactivity respond better to a low MPH dose, whereas those without hyperactivity but with higher inattention respond better to a higher dose (22, 23). Baseline severity of aggression, anxiety, and abnormal fear processing may play an important role in the modulation of the behavioral outcome observed in children diagnosed with these disorders.

Animal models enable us to study the effect of pharmacological interventions on a range of behaviors in a controlled environment. Most preclinical studies have shown no effect, some others anti-aggressive effects of MPH (24–26), and other studies observed anxiolytic effects of MPH (26). In addition, MPH was found to improve fear extinction when administered before or immediately following extinction of contextual fear (27). However, no preclinical study has investigated the effect of MPH dose on aggression, anxiety, and fear processing/extinction in the same animal model.

Previous research by our group has focused on the BALB/cJ mouse, an animal model that shares phenotypic traits similar to those seen in ADHD and CD, including inattention, increased aggression, and reduced social behavior (28–30). Furthermore, reversal learning with conditioned punishment revealed increased reward motivation and decreased punishment sensitivity to be present in BALB/cJ mice, which could point towards altered DA signaling as well (Jager et al., submitted). The present study was designed to examine the effect of two doses of acute MPH administration [3 and 10 mg/kg intraperitoneal (i.p.)] on the behavior of BALB/cJ mice in order to answer the following questions: (1) Does MPH affect anxiety and fear extinction? (2) Does MPH affect social behavior and aggression, and if so, is this dose dependent? The answers to these questions will provide insights into the regulation of monoamine tone in the brain and its influences on anxiety and aggression.

## Methods

### Animals

Eight-week-old male BALB/cJ mice (*n* = 36) (The Jackson Laboratory, United States) and six-week-old C57BL/6J male mice (*n* = 36) (Charles River, Germany) were used in this study. Exclusively male mice were used in order to eliminate possible variation caused by the estrous cycle of female mice. Upon entry, all mice were provided with a unique tail number. All mice were housed at the institutional animal facility in an individually ventilated cage (type 2L, Tecniplast S.p.A., Buguggiate, Italy) with an igloo as environmental enrichment and had *ad libitum* access to water and food. BALB/cJ mice were housed individually, and C57BL/6J mice in groups of six mice under a reversed light/dark cycle (12/12 h) in a ventilated cabinet, Scantainer (Scanbur, Karlslunde, Denmark) with sunset at 7.00 am at a constant temperature of 24 ± 1°C. All experimental procedures were approved by the Committee of Animal Experiments of the Radboud University Medical Center (project number: DEC2013-235), Nijmegen, Netherlands.

### Drug Administration

MPH (Brocacef, Maarssen, Netherlands) was prepared freshly every morning of the testing and dissolved in 0.9% saline. MPH (3 or 10 mg/kg of body weight) or vehicle (0.9% saline) was delivered to BALB/cJ mice (*n* = 12 per condition) 20 min prior to testing by intraperitoneal (i.p.) injection. Animals were returned to their housing case until the time of testing. Injection sides were alternated in order to reduce tissue damage. The dosage of MPH administered to the mice was based on previous animal studies that have investigated locomotor activity and cognition in rodents (in details explained by 31). The dose of 3 mg/kg resembles the therapeutic window of treatment of ADHD in humans, while the 10 mg/kg dose may reflect more the recreational use of MPH (32, 33). Drug administration was randomized, and experimenters were blinded to the test conditions. The blinding code was broken after the completion of the data analysis.

### Behavioral Tests

**Figure 1:**
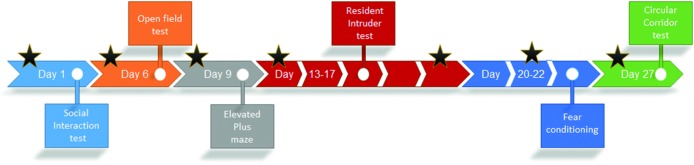
Schedule of experimental testing. Timeline of the different experiments using BALB/cJ mice treated with 3 mg/kg methylphenidate (MPH) i.p., 10 mg/kg MPH i.p., or vehicle (n = 12 per group). Experiments were performed in three cohorts of four animals per condition in order to keep testing within the first hours after darkness. Drug injections were given 20 min prior to the test and are indicated by stars. During the resident–intruder test, animals were injected on day 1 and day 5, whereas during fear conditioning, animals were only injected on day 2.

All behavioral tests were carried out in the same experimental room. The experimental schedule can be found in **Figure 1**. Animals were transported in their cage to the room 1 h prior to testing. The order of testing of the mice was randomized for each of the behavioral experiments. All experiments were performed in the dark phase under red-light conditions, with the exception of the elevated plus maze, which was performed in the dark phase under dim-light conditions. No experiments were performed within the first hour after the light/dark transition. To enable testing within the first hours of darkness, the experiments were performed in three cohorts (3 × *n* = 4 per treatment group). Only the last cohort (*n* = 4 per treatment group) was tested in the circular corridor. All animals were given 1 week to acclimatize to the animal housing facility prior to behavioral testing.

#### Social Cognition Test

The effect of MPH on social behavior was assessed in the social cognition test on day 1 of the experiment. Two wire-mesh cylinders with large open ventilation holes (*l* × *w* × *h* 10 × 10 × 11 cm) were placed upside-down in a clear observation cage (dimensions 43 × 50 cm) with corn bedding material on the floor, as shown in **Figure S1**. An unfamiliar C57BL/6J mouse was placed under a randomly assigned cylinder. Subsequently, a BALB/cJ mouse was placed in the middle of the cage, and behavior was recorded for 5 min using a high-speed infrared camera (GigE, Basler AG, Ahrensburg, Germany). MediaRecorder (Noldus, Wageningen, Netherlands) was used to record these movies. Time spent in the social zone, which contained the cylinder with the C57BL/6J mouse, was compared to the time spent in the non-social zone. The frequency and time that the animals spent sniffing the social and non-social cylinder were analyzed as well. These behaviors were manually scored with Observer XT (Noldus, Wageningen, Netherlands).

#### Open Field

To assess the effect of MPH on anxiety, an open-field test was performed on day 6. Locomotion activity was quantified in a 55 × 55 × 36 cm activity chamber (made by our laboratory). The animals (*n* = 12 per group) were placed in the center of the field where locomotion activity was then recorded for 5 min. The arena was divided into four quadrants in which the connected center points of all quadrants formed the center of the field and measured 27.5 × 27.5 cm. The total time in the center zone, outside the center and time spent near the walls, was measured as well as the frequency of center visits. In addition, the latency to leave the center was used as an indication of non-explorative behavior (immobility). Reduced frequency of center visits, velocity, and distance traveled were used as indications of locomotor activity and anxiety behavior. The arena was cleaned with 70% alcohol between tests. Videotapes of the locomotion activity were examined using EthoVision XT9 (Noldus, Wageningen, Netherlands).

#### Elevated Plus Maze

Anxiety and explorative behavior were assessed in the elevated plus maze 20 min after drug administration on day 9. The elevated plus maze (Noldus, Wageningen, Netherlands) consisted of two open (36 cm × 6 cm) arms and two closed (36 cm × 6 cm, modeled with 15-cm-high walls) arms emanating from a common central platform (6 cm × 6 cm) and was placed 60 cm above the ground. At the start, animals were placed at the junction of the open and closed arms with the head facing the closed arms. The behavioral test was performed in the dark phase under dim-light conditions, and exploratory behavior was videotaped with a high-speed camera (25 frames per second) (GigE, Basler AG, Ahrensburg, Germany) for 5 min. EthoVision XT9 software (Noldus, Wageningen, Netherlands) was used to track the activity patterns. The times spent in the open and closed arms were examined as a time ratio (RT). The RT is the time spent in the open arms (TO)/total time spent in both closed (TC) and open arms (TO): RT = TO/(TO + TC). In addition, the frequency of transition between the arms, the total distance traveled, and the velocity were measured. Between each animal, the maze was cleaned with 70% ethanol and dried before testing the next animal.

#### Resident–Intruder Paradigm

The resident–intruder paradigm was performed in order to assess territorial aggression on five consecutive days. The experiment started on day 13 and lasted until day 17, and testing took place in the housing cages of BALB/cJ mice that had not been cleaned after arrival. On each testing day, an unfamiliar C57BL/6J intruder mouse was encountered, which was randomly assigned to a resident for each interaction. All animals, both resident and intruder, were tested once a day. The housing cage of the resident was used as the interaction area, which was placed in front of an infrared high-speed camera (25 frames per second) (GigE, Basler AG, Ahrensburg, Germany). A transparent Plexiglas screen was placed in the middle of the cage, to prevent direct interaction between animals but to enable visual, auditory, and olfactory perception. The intruder mouse was placed at the other side of the plastic screen for a period of 5 min. Hereafter, the screen was removed, and the interaction was videotaped for 5 min. After the test, the intruder was removed from the cage, and both animals were weighed and checked for wounds. Treatment with vehicle or MPH was given 20 min prior testing, but only on the first and last days of the resident–intruder paradigm. The frequency of attacks and bites and the latency to the first attack were analyzed manually for all interaction days. A broader range of the behavioral repertoire (e.g., bites, attacks, lateral threats, and tail rattles as described by 34, 35) was scored on the MPH-treated days (day 1 and day 5). An attack latency of 300 s was taken in case no attack occurred within the 5-min interaction window.

#### Mixed-Cue Fear Conditioning

Fear conditioning was performed in Bussey-Saksida Mouse Touch Screen Chambers (Campden Instruments Ltd, Loughborough, United Kingdom) on days 20–22. An aversion stimulus dual shocker (Kinder Scientific, Poway, California, United States) connected to the chambers was used to provide manual shocks to the animals *via* a grid floor. On the first day, in the “cue conditioning” phase, animals were placed individually into the fear-conditioning arena and were habituated for 3 min. After habituation, the cue conditioning started with an acoustic conditioned stimulus (CS) (2 kHz), which lasted for 30 s, which was accompanied by an unconditioned stimulus (US) of electric foot shock (0.7 mA) during the last 2 s of the CS. This cue conditioning was repeated three times with an inter-trial interval of 95 s. On the second day, in the “cue extinction” phase, animals were injected with either MPH or vehicle 20 min prior to the test. Mice were placed in the same box as the day before and were again habituated for 3 min before the trials started. Fifteen trials were given, exclusive of the CS, with a duration of 30 s and an inter-trial interval of 10 s between them. No US stimulus was given in the cue extinction. On the third day, the protocol of the second day was repeated, only without drug administration. Between and after each test, the fear-conditioning area was cleaned with 70% ethanol. Freezing during the 15 cue extinction trials was quantified manually by Observer XT software (Noldus, Wageningen, Netherlands). As an additional measure, the amount of beam breaks during the habituation on all 3 days and the beam breaks during the 15 cue extinction trials (on day 2 and day 3) were analyzed with ABET II Software for Touch Screens (Lafayette Instruments Company, Lafayette, Indiana, USA).

#### Circular Corridor

Locomotor activity of the animals after treatment with MPH or vehicle (*n* = 4 per group) was measured in the circular corridor test. In order to keep testing within the first hours after darkness, only three animals were tested per morning session, and animals were randomly assigned to the testing days on either day 27, 28, 30, or 36. This setup consists of two Plexiglas cylinders; the outer cylinder had a diameter of 24 cm and was 20 cm in height, and the inner cylinder had a diameter of 11.5 cm and was 15 cm in height, which creates a corridor of 6.25 cm in width. Each animal was free to explore the circular corridor for 60 min, without prior habituation, 20 min after the injection. The exploration pattern was recorded with a high-speed infrared camera (25 frames per second) (GigE, Basler AG, Ahrensburg, Germany) placed above the setup. After each BALB/cJ, the setup was cleaned with 70% ethanol. EthoVision XT9 software (Noldus, Wageningen, Netherlands) was used to analyze the distance that was traveled within the total testing period and within the first 15 min, which was thought to be the most effective period of MPH, since the DA increase by MPH decreases 40 min after the *i.p.* injection (32).

### Data Analysis and Statistics

An *a priori* power analysis was conducted with an expected effect size of 0.60, an *α* of 0.05, and *β* of 0.80, which predicted that a minimum of 10 mice per condition was required. Based on prior experience, we predicted that approximately 20% of the animals had to be excluded from further analysis due to behavioral reasons or other complications caused by the injections. For these reasons, we used 12 animals per treatment group. All data were analyzed using IBM SPSS Statistics (version 24.0, Chicago, USA). Normality of the data was assessed with the Kolmogorov–Smirnov test. Data that were normally distributed were analyzed by paired Student *t*-tests or (repeated measures) analysis of variance (ANOVA), followed by Bonferroni corrected *post hoc t*-tests. Nonparametric Mann–Whitney tests, Wilcoxon signed rank tests, and Kruskal–Wallis tests were used for non-normally distributed data. The Dunn *post hoc* method was performed to correct for multiple nonparametric comparisons. Relationships within the data (elevated plus maze: ratio open/closed arms; open field: number of center visits; resident–intruder test: number of bites, number of attacks; social interaction test: time spent near social cylinder; mixed-cue fear-conditioning test: time spent freezing; total number of beam breaks; and circular corridor: total distance travelled) were assessed using Pearson correlations. Outliers in the data were excluded by Tukey’s method, namely, when diverging more than 1.5 × interquartile range different from the median (36). All statistical tests were two-sided with a significance level of *p* < 0.05, indicating statistical significance. GraphPad PRISM (version 5.03, GraphPad Software Inc., La Jolla, US) software was used to output data into images.

## Results

### MPH Suppresses Anxiety-Related Behavior

**Figure 2:**
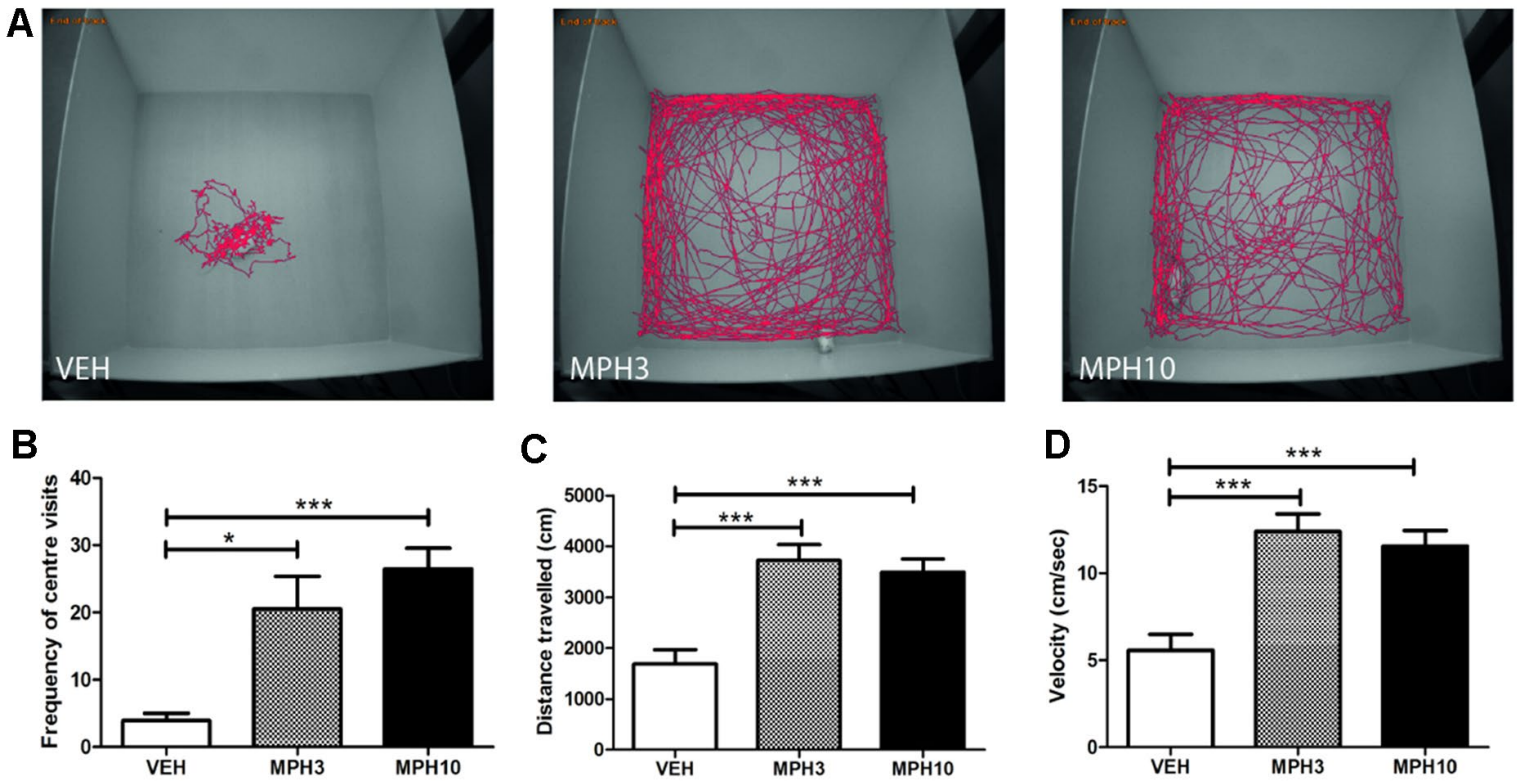
Open-field experiment. **(A)** Typical representations of exploratory patterns of BALB/cJ mice in open field. While the vehicle remains most of the time at the start position, the methylphenidate (MPH)-treated animals explore the whole field, and **(B)** MPH increased the number of center visits and **(C)** the distance traveled as well as **(D)** the velocity. Abbreviations: VEH, vehicle; MPH3, 3 mg/kg i.p. methylphenidate; MPH10, 10 mg/kg i.p. methylphenidate. N = 12 per group, *p < 0.05, ***p < 0.001.

The open-field test and the elevated plus maze were performed in order to assess the effect of MPH on anxiety and explorative behavior in BALB/cJ mice. Similar to a previous report, BALB/cJ mice treated with vehicle exhibit a long period of immobility in the starting position, which was the center of the field (30). Both 3 and 10 mg/kg *i.p.* MPH increased the frequency of visits to the center (*H*_2_ = 15.121, *p* = 0.001, VEH vs MPH3: *p* = 0.016; VEH vs MPH10: *p* < 0.001) (**Figures 2A, B**). Additionally, the distance traveled and the velocity increased significantly compared to those in vehicle-treated animals (**Figures 2C, D**).

**Figure 3:**
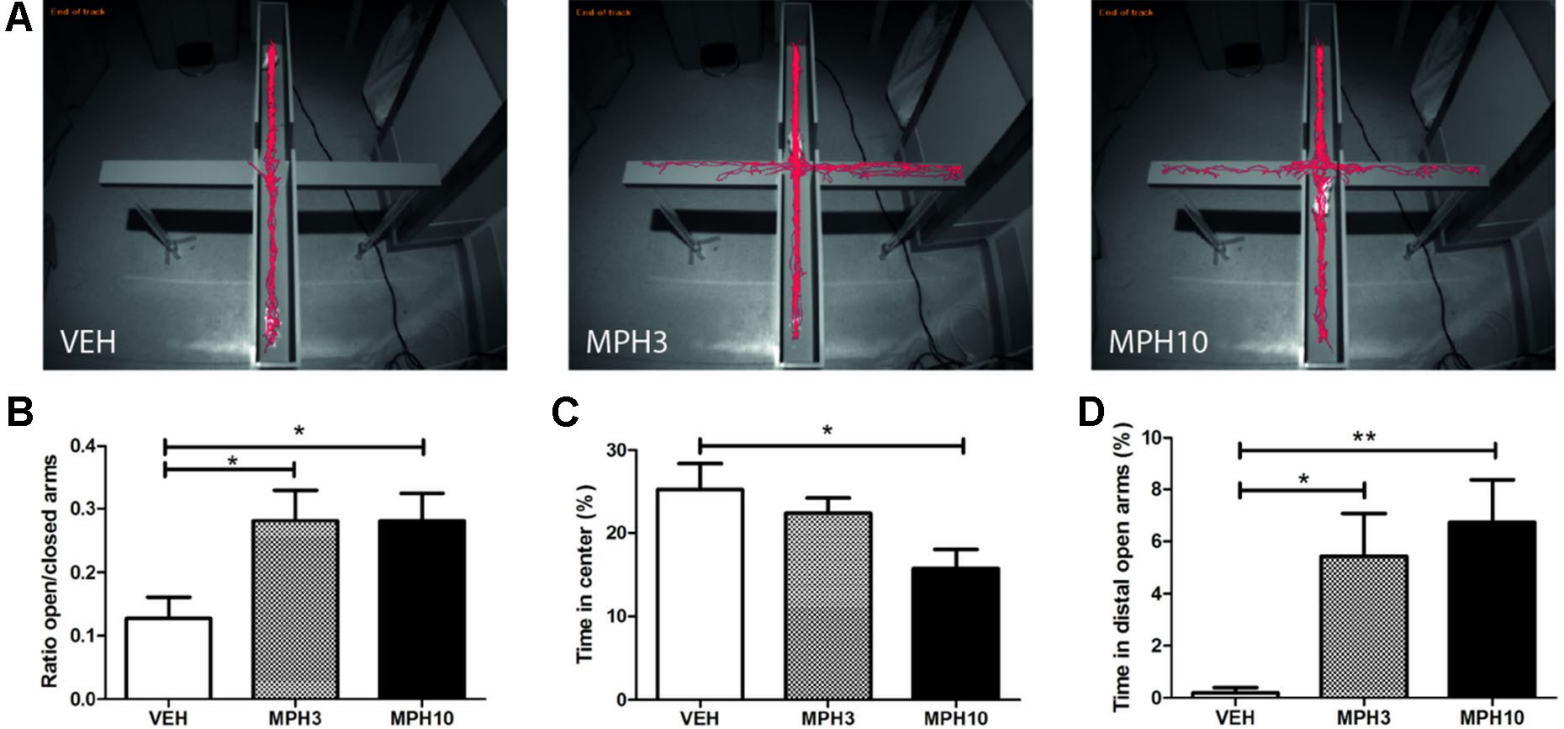
Elevated plus maze. **(A)** Typical representations of exploratory patterns of BALB/cJ mice on the elevated plus maze. While the vehicle remains in the closed arms for most of the time, the methylphenidate (MPH)-treated animals explore the open arms. **(B)** MPH increased the time spent on the open arms as a ratio of the time spent on both the closed and open arms. **(C)** The vehicle-treated animals stay in the center of the maze. **(D)** The MPH-treated animals spent more time in the distal parts of the open arms. Abbreviations: VEH, vehicle; MPH3, 3 mg/kg i.p. methylphenidate; MPH10, 10 mg/kg i.p. methylphenidate. N = 12 per group, *p < 0.05, **p < 0.01.

Similar findings were observed in the elevated plus maze, where, as shown in **Figures 3A, B**, a significant difference was found in the ratio between the duration spent in the open and closed arms of the maze between vehicle- and MPH-treated groups. After administration with either dosage of MPH, mice spent significantly more time in the open arms compared to mice treated with vehicle (*H*_2_ = 0.407, *p* = 0.816; VEH vs MPH3: *p* = 0.038; VEH vs MPH10: *p* = 0.040; MPH3 vs MPH10: *p* = n.s.). BALB/cJ mice treated with vehicle spent more time on the central platform of the maze compared to mice treated with MPH (*F*_(2,33)_ = 4.4506, *p* = 0.019; VEH vs MPH3: *p* = n.s.; VEH vs MPH10: *p* = 0.015, MPH3 vs MPH10: *p* = n.s.) (**Figure 3**). To examine anxiolytic effects of MPH, the duration spent in the distal open arms was examined, as a gauge for their exploratory/anxiety behavior. MPH significantly increased the time spent in the distal open arms of the elevated plus maze, in both treatment groups compared to the vehicle group (*H*_2_ = 10.619, *p* = 0.005; VEH vs MPH3: *p* = 0.026; VEH vs MPH10: *p* = 0.002, **Figure 3**). Additionally, the velocity and distance moved were significantly different between all three treatment groups. Compared to vehicle, administration of MPH increased the velocity [*F*_(2,33)_ = 10.12, *p* ≤ 0.001; VEH vs MPH3: *p* = 0.037; VEH vs MPH10: *p* ≤ 0.001; MPH3 vs MPH10: *p* = n.s., **Figure S2A**] and the distance traveled in a dose-dependent manner [*F*_(2,33)_ = 10.209, *p* ≤ 0.001; VEH vs MPH3: *p* = 0.040; VEH vs MPH10: *p* ≤ 0.001; MPH3 vs MPH10: *p* = n.s., **Figure S2B**). Previous studies showed increased locomotor activity by administration of MPH (37). To examine the possibility that the increased distance traveled, in both the open-field test and the elevated plus maze, was the result of hyperactivity caused by MPH administration, a circular corridor test was performed. Locomotor activity was measured during the first 15 min as a measure of treatment reactivity to the novel environment. A significant difference has been found in the traveled distance between the 10 mg/kg MPH and vehicle treatment groups [*F*_(2,8)_ = 5.519, *p* = 0.031; VEH vs MPH3: *p* = n.s.; VEH vs MPH10: *p* = 0.040; MPH3 vs MPH10: *p* = n.s., **Figure S3**). While the 3 mg/kg MPH-treated group did not differ in explorative behavior compared with the vehicle group, we cannot completely rule out a hyperactivity effect on the anxiety measures given the limited number of observations.

### Improved Fear-Extinction Learning by MPH

**Figure 4:**
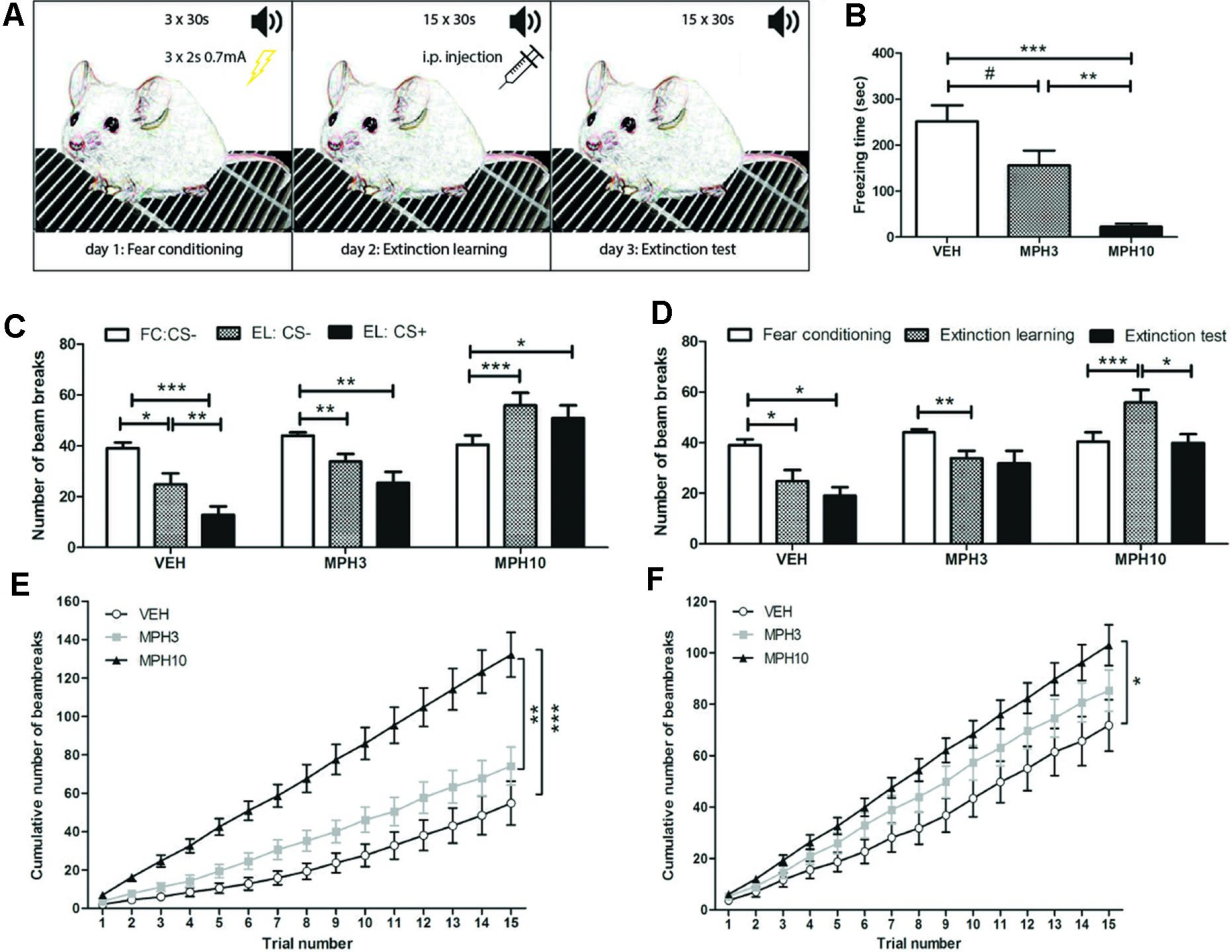
Mixed-cue fear-conditioning test. **(A)** Overview of the test procedure. On the first day (fear conditioning), animals received three sessions of 30 s of an auditory cue, of which the last 2 s was paired with 0.7 mA foot shock. On day 2, the extinction learning, the auditory cue was presented during 15 trials of 30 s without the shock. Animals were injected with either MPH or vehicle 20 min before this task. On the third day, the extinction test, the auditory cue was presented during 15 trials of 30 s each. **(B)** The time spent freezing on the extinction learning day decreased with the methylphenidate (MPH) dose. **(C)** The number of beam breaks is presented during the habituation phases (CS−) of the fear conditioning (FC) and extinction learning (EL) and during the presentation of the auditory cue (CS+) during EL. **(D)** The number of beam breaks of the habituation phases is compared between all three testing days. **(E)** The graph represents the cumulative number of beam breaks on the extinction learning day and **(F)** extinction test day. Abbreviations: FC, fear conditioning; EL, extinction learning, i.p., intraperitoneal; CS−, conditioned stimulus absent; CS+, conditioned stimulus present; VEH, vehicle; MPH3, 3 mg/kg i.p. MPH; MPH10, 10 mg/kg i.p. MPH. N = 12 per group, ^#^p < 0.010; *p < 0.05; **p < 0.01; ***p < 0.001.

Extinction of conditioned fear was based on the reduction in the response according to the repetitive presentation of a tone (CS) in the absence of the foot shock (US). We assessed the effect of MPH on mixed-cue fear conditioning in BALB/cJ mice. Drug treatment was given prior to fear extinction learning as depicted in **Figure 4**. During extinction learning, administration of MPH decreased the freezing time in mice treated with 3 and 10 mg/kg MPH compared to vehicle [*F*_(2,33)_ = 17.275, *p* ≤ 0.001; VEH vs MPH3: *p* = 0.059; VEH vs MPH10: *p* = < 0.001; MPH3 vs MPH10: *p* = 0.005, **Figure 4**]. As an additional measure of activity, we measured the number of infrared beams, located at the front and the back of the chamber, that were crossed by the animals. No significant differences were found in the activity during the habituation phase before conditioning between the treatment groups, indicating no prior group differences in anxiety or locomotor activity (**Figure S4**). During extinction learning, a significant difference in baseline locomotor activity was found between the highest dose of MPH and the other groups [*F*_(2,33)_ = 14.41, *p* ≤ 0.0001; VEH vs MPH3: *p* = 0.866; VEH vs MPH10: *p* ≤ 0.0001; MPH3 vs MPH10: *p* = 0.001], in which the animals treated with 10 mg/kg MPH demonstrated an increased number of beam breaks (**Figures 4** and **S4**). The vehicle group (*t*_24_ = 2.934, *p* = 0.014) and the 3 mg/kg MPH group (*t*_18_ = 3.624, *p* = 0.007) showed a reduction in the number of beam breaks when comparing activity during the habituation phase of the conditioning and extinction phase, while in the 10 mg/kg MPH group, an increased number in bream breaks was found (*t*_24_ = −4.983, *p* ≤ 0.001) (**Figures 4** and **S4**). In addition, during extinction learning, only the vehicle group showed significantly reduced activity when the CS was presented (*t*_24_ = 3.970, *p* = 0.002), as is shown in **Figure 4**. A long-lasting effect of the fear conditioning was seen during the extinction test in the vehicle-treated animals, in which the number of beam breaks was still significantly reduced when compared to the habituation phase of the conditioning day (*t*_24_ = 2.382, *p* = 0.036); this was not observed in the MPH-treated mice (**Figure 4**). In addition, administration of 10 mg/kg of MPH increased the total number of beam breaks over 15 CS+ sessions significantly during both extinction learning [*F*_(2,33)_ = 16.880, *p* ≤ 0.001; VEH vs MPH3: *p* = n.s.; VEH vs MPH10: *p* ≤ 0.001; MPH3 vs MPH10: *p* = 0.001, **Figure 4**] and the extinction test [*F*_(2,33)_ = 3.702, *p* = 0.035; VEH vs MPH3: *p* = n.s.; VEH vs MPH10: *p* = 0.031, MPH3 vs MPH10: *p* = n.s., **Figure 4**] compared to vehicle, indicating a long-lasting effect of MPH on fear extinction.

### MPH Affects Social Interest Dose-Dependently

**Figure 5:**
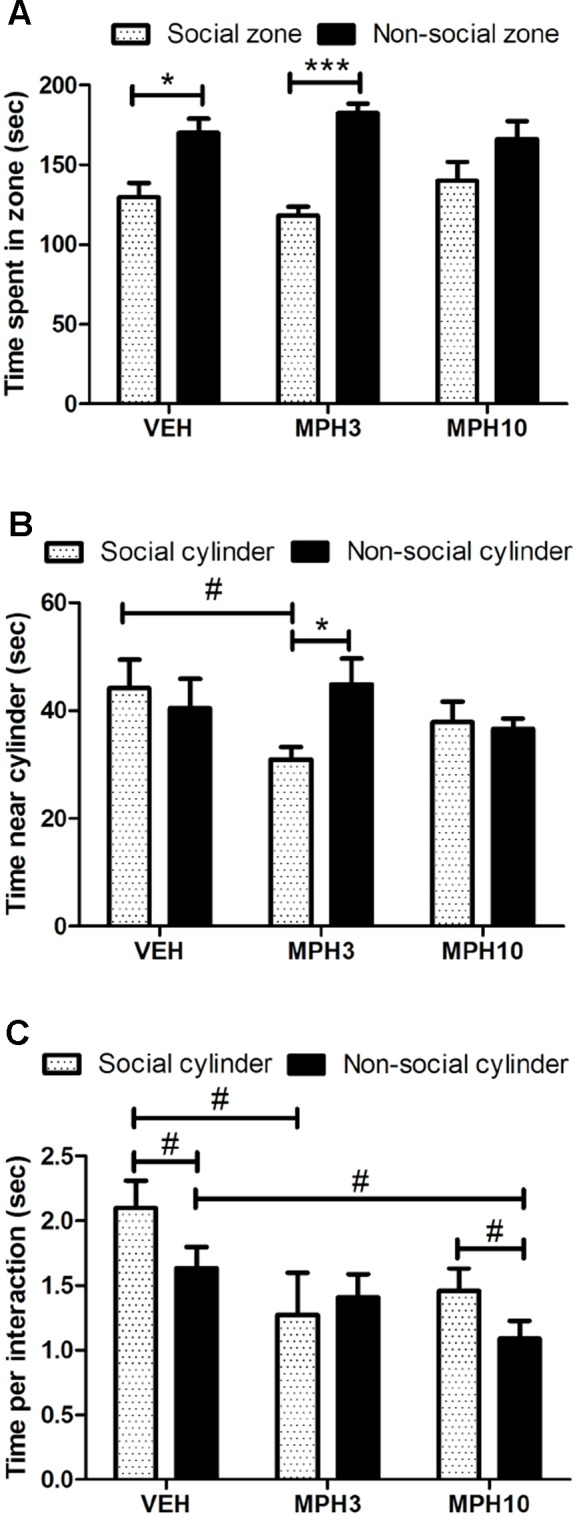
Social interaction test. **(A)** BALB/cJ mice treated with vehicle and 3 mg/kg i.p. methylphenidate (MPH) show a preference for the non-social zone. **(B)** The dose of 3 mg/kg i.p. MPH increased the preference for the non-social cylinder in comparison to the social cylinder and the vehicle-treated mice. **(C)** Both the vehicle-treated and 10 mg/kg i.p. MPH-treated mice show a longer interaction time with the social cylinder, while this is not the case for the 3 mg/kg group. Abbreviations: VEH, vehicle; MPH3, 3 mg/kg i.p. MPH; MPH10, 10 mg/kg i.p. MPH. N = 12 per group, ^#^p < 0.10; *p < 0.05; ***p < 0.001.

Previous observations have indicated decreased social interaction in BALB/cJ compared to C57BL/6J (28). Therefore, we have investigated whether MPH has an effect on social behavior using the social cognition test and the resident–intruder paradigm. The social cognition test showed that all groups have increased interest for the zone without the unfamiliar animal, the so-called non-social zone (VEH *t*_11_ = −2.258, *p* = 0.045; MPH3 *t*_10_ = −5.561, *p* < 0.001; MPH10 *t*_11_ = −1.168, *p* = 0.267; **Figure 5**). However, if we focus on the time that the animals have investigated the cylinder itself, there is no difference between the time spent near the social and non-social cylinders for the vehicle-treated animals (*t*_11_ = 0.479, *p* = 0.641) and animals treated with 10 mg/kg MPH (*t*_10_ = 0.340, *p* = 0.741). In contrast, animals treated with 3 mg/kg MPH have a preference for the non-social cylinder (*t*_9_ = −2.722, *p* = 0.024) (**Figure 5**). In addition, the vehicle-treated animals spend more time per interaction with the social cylinder in comparison to the non-social cylinder (**Figure 5**). In addition, it was found that the time per interaction with the social cylinder was also decreased for the animals that received MPH in contrast to animals that received vehicle [*F*_(2,33)_ = 3.189, *p* = 0.055; VEH vs MPH3: *p* = 0.065; VEH vs MPH10: *p* = 0.214, **Figure 5**]. While both the vehicle group and the 10 mg/kg MPH group spent a longer time per interaction with the social cylinder in comparison to the non-social cylinder, this was not found in the mice treated with 3 mg/kg MPH (**Figure 5**).

### MPH Dose-Dependently Produces Opposing Effects on Aggression

**Figure 6:**
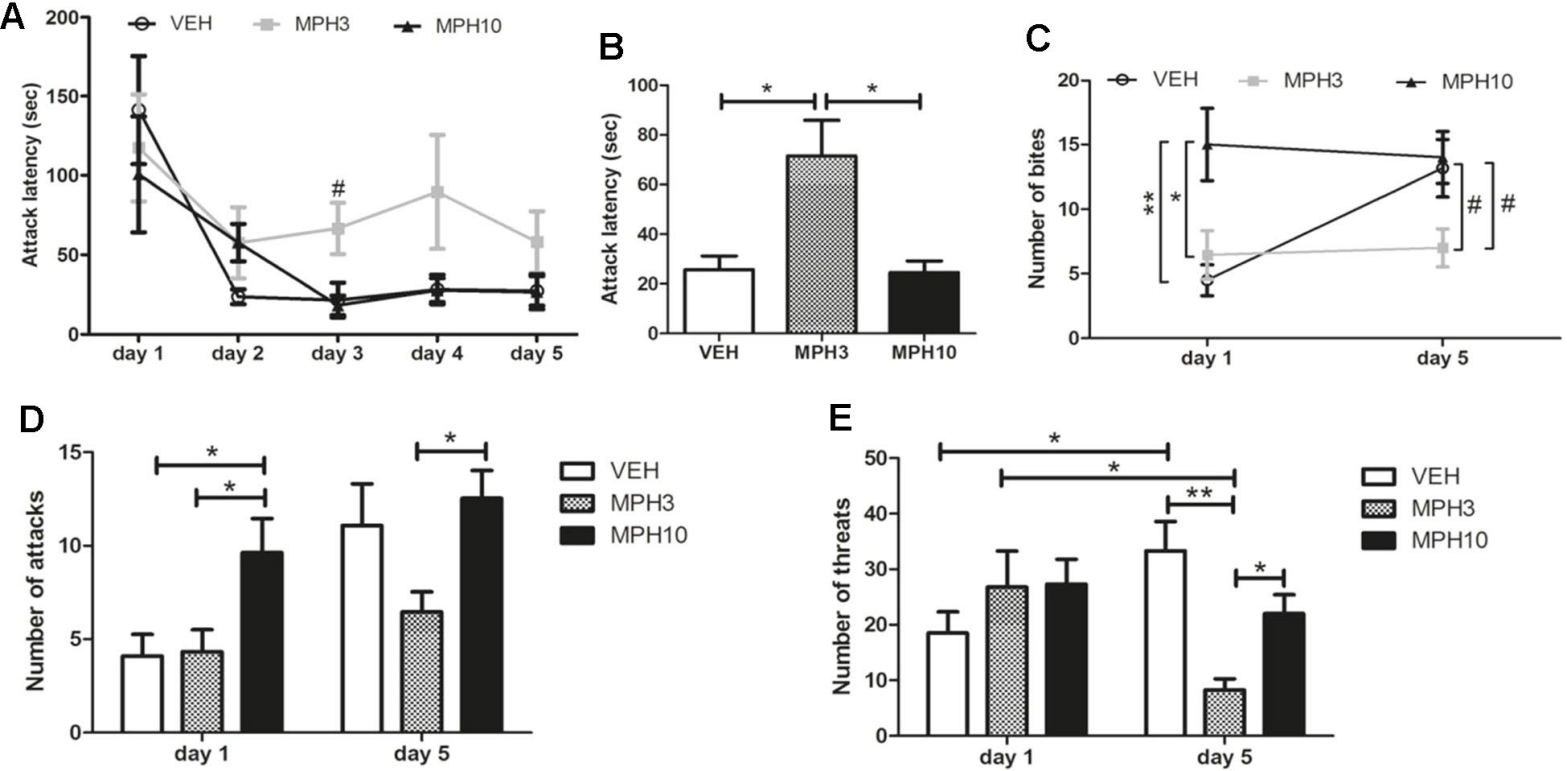
Resident–intruder test. **(A)** The attack latency over the five testing days did not significantly differ between the treatment groups. **(B)** The average attack latency over the last 3 days is significantly higher in the 3 mg/kg methylphenidate (MPH)-treated group. **(C)** The number of bites increased after administration of 10 mg/kg MPH on the first day. While the number of bites increased over time for the vehicle-treated BALB/cJ mice, the number of bites remained low for the group treated with 3 mg/kg MPH. **(D)** The group treated with 10 mg/kg MPH had an increased number of attacks on day 1 and on day 5 in comparison to the 3 mg/kg MPH-treated group. **(E)** Threat behavior significantly reduced in the group treated with 3 mg/kg MPH, while this increased for the vehicle-treated group. VEH, vehicle; MPH3, 3 mg/kg i.p. MPH; MPH10, 10 mg/kg i.p. MPH. *N* = 12 per group, #*p* < 0.10; *p < 0.05; **p < 0.01.

The resident–intruder paradigm was performed to assess aggression in BALB/cJ mice treated with MPH or vehicle. The test was performed for five successive days, in which BALB/cJ mice were only treated the first and last days of the test with MPH or vehicle 20 min prior testing (see **Figure 1**). As displayed in **Figure 6**, no significant difference was found between the attack latencies of BALB/cJ of the different treatment groups. For all groups, attack latencies decreased on the second day of the resident–intruder paradigm compared to the first day, which is considered as normal behavioral response, and aggression is stabilized after 3 days (35). Although there was no significant difference between groups when each day was compared separately, it was found that the average attack latency of the last 3 days was longer for the 3 mg/kg MPH-treated animals (*H*_2_ = 6.273, *p* = 0.043) (**Figure 6B**). **Figure 6C** shows the number of bites on both injection days (day 1 and day 5). Whereas we see an increase in the number of bites from days 1 to 5 in the vehicle-treated animals (*t*_18_ = −3.091, *p* = 0.013), the level of aggression stays at the level of day 1 for the 3 mg/kg (*t*_16_ = 0.555, *p* = 0.594) and 10 mg/kg (*t*_22_ = 0.263, *p* = 0.798) MPH-treated animals. An increased number of bites is observed in the 10 mg/kg MPH on day 1 [*F*_(2,30)_ = 6.789, *p* = 0.004; VEH vs MPH10: *p* = 0.006; MPH3 vs MPH10: *p* = 0.024) in comparison to the other groups (**Figure 6C**). On day 5, both the vehicle-treated animals and the 10 mg/kg MPH-treated animals have a higher number of bites in comparison to the group treated with 3 mg/kg MPH (*F*_(2,31)_ = 3.474, *p* = 0.044; VEH vs MPH3: *p* = 0.117; MPH3 vs MPH10: *p* = 0.061) (**Figure 6C**). A similar pattern is found for the number of attacks, as is shown in **Figure 6D**. This number of attacks increases for the vehicle group (*t*_20_ = −2.691, *p* = 0.023), whereas it remains at the level of day 1 for both the 10 mg/kg MPH (high number of attacks) and the 3 mg/kg MPH (low number of attacks). Interestingly, in the 3 mg/kg MPH group, the number of threats reduced over time (*Z*_24_ = −2.491, *p* = 0.013), while the number of threats increased for the vehicle-treated animals (*Z*_24_ = −2.357, *p* = 0.018) (**Figure 6E**). On day 5, the number of threats in the 3 mg/kg MPH group is significantly lower (*H*_2_ = 14.650, *p* = 0.001) than that in the vehicle group (*p* = 0.002) and the 10 mg/kg MPH group (*p* = 0.008) on day 5. At the first day of the resident–intruder test, there is no significant difference between the number of threats observed in all three groups, which indicates that the elevated level of aggression seen in the 10 mg/kg MPH group is accompanied by less threat behavior.

## Discussion

The current study assessed the effect of MPH on anxiety, fear extinction, and aggressive behavior in the BALB/cJ mouse, as a model for these behavioral characteristics in relation to ADHD and CD. Our previous research has shown that BALB/cJ mice exhibit increased aggression, increased anxiety, and antisocial behavior (28–30), which makes this mouse strain an ideal model to answer the following questions: (1) Does MPH affect anxiety and fear extinction? (2) Does MPH affect social behavior and aggression, and if so, is this dose dependent? A clear overview of the behavioral outcome can be found in **Table 1**.

**Table 1:** Behavioral effects of different dosages of methylphenidate in BALB/cJ mice.

Drug	Behavior				
Methylphenidate dose	Locomotion	Anxiety	Social interest	Fear extinction	Aggression
Low (3 mg/kg)	–	↓	↓	↑	↓
High (10 mg/kg)	↑	↓	–	↑↑	↑

### Anxiolytic and Fear Extinction-Improving Effects of MPH in a Dose-Dependent Manner

In BALB/cJ mice, treatment with both the 3 and 10 mg/kg dose had an anxiolytic action in the open field and in the elevated plus maze. This may reflect changes in state anxiety. Opposite results were obtained in a study in C57BL/6J mice, which became more anxious after MPH treatment (38, 39). C57BL/6J mice are in general less anxious than BALB/cJ mice (40), so this result may indicate that MPH can have different effects in different (high and low anxiety) rodent strains, which may be a consequence of differential drug-genetic background effects. Administration of 2.7 mg/kg MPH *i.p.* to Wistar rats exposed to unpredictable chronic stress decreased the immobility compared to saline-injected rats after fear conditioning (41). Similarly, we found that administration of 3 and 10 mg/kg *i.p.* MPH reduced immobility upon the US and improved extinction, measured by the number of beam breaks and the time spent freezing. Improved extinction learning was even visible 24 h after the actual injection of 10 mg/kg *i.p.* was given (extinction test phase), indicating a long-lasting effect of MPH on fear retrieval, which suggest changes in trait anxiety and fear processing. In agreement with our findings, a study in C57BL/6 mice showed that administration of MPH before or immediately following extinction of contextual fear enhances extinction retention, with also long-lasting effect of the 10 mg/kg *i.p.* dose (27). Still, it needs to be considered that these long-lasting effects of MPH may also be due to changes related to the circadian rhythm of the subjects. We have repeatedly administered MPH to the animals, and to prevent accumulation of the drug, we scheduled a minimum of 3 days between injections. However, we cannot be certain that the effect of the drug from preceding administration did not induce some longer-lasting biological change, for example, gene expression that may affect subsequent tests.

It was found that administration of MPH after the extinction learning was effective, indicating that the timing of the injection has a large influence on the behavioral outcome (27, 42). Given the highly anxious phenotype of BALB/cJ mice (30), we could argue that MPH reduced both the state and trait anxiety during fear extinction and prevented fear retrieval. Since we observed long-lasting effects of the 10 mg/kg *i.p.* dose, this may indicate that the initial association between the CS and US has been diminished and may reflect an alteration of the underlying trait. The clinical implications of MPH utility on fear processing need to be more fully explored but may involve complex interactions with genotype and environmental triggers, so caution should be taken in extrapolating the ability of MPH to reduce immobility/freezing and improve fear extinction processing in this model. Moreover, while reducing anxiety may play a part of the anti-aggressive response of low-dose MPH, it is not the only possible explanation, especially as both doses reduce anxiety but only the lower dose is anti-aggressive. The role of anxiety in regulating aggression in BALB/cJ mice is worth following up in directed studies. We have previously published that GABAergic inhibition is diminished in the anterior cingulate cortex (ACC) of these mice (relative to BALB/cByJ mice) (30), a brain region also implicated in anxiety, so anti-anxiety effects of MPH in this model should not be fully discounted as it may reduce defensive behavior. Investigation of the effects of MPH on GABAergic transmission in the ACC of BALB/c substrains would be useful to ascertain a mechanism of action for the behavioral effects reported here.

We observed that MPH increased locomotor activity in both the open field and elevated plus maze. While there was no difference between the distance traveled between the 3 and 10 mg/kg *i.p.* MPH-treated BALB/cJ mice in the open field, a dose-dependent effect was observed in the elevated plus maze. Additional testing to clarify any locomotion-related effect demonstrated that in the circular corridor paradigm, only the higher 10 mg/kg dose of MPH was associated with hyperactivity in BALB/cJ mice. While no difference was observed between the vehicle-treated animals and those administered 3 mg/kg MPH, we cannot completely rule out an effect caused by hyperactivity in the animals treated with the 3 mg/kg MPH because of the low sample size used in this paradigm. Previous studies that administered MPH to rodents also showed a dose–response-dependent increased locomotor activity in which the effect was clearest in the high-dose group (25, 43, 44). However, in our experimental setting, it needs to be considered that the fear-conditioning test, a week prior to the corridor test, may have influenced the behavioral outcome in the test.

While it is clear that both the low and high doses of MPH increase motor activity in the elevated plus maze, this is not correlated with the anxiety metrics in the elevated plus maze, nor was this effect observed in the open-field test. The correct interpretation of any MPH-induced hyperactivity is important as it can confound the interpretation of other behavioral results. Since anti-aggressive effects were observed at 3 mg/kg *i.p.* and increased aggression at the 10 mg/kg *i.p.* dose of MPH, it cannot be concluded that any effect on locomotion at these doses altered outcome on aggression. Follow-up experiments should test dosages lower than 3 mg/kg *i.p.* that still have aggression-reducing effects without increasing locomotor activity. Furthermore, we demonstrate that the high dose of MPH 24 h later continues to exert a restoration of fear extinction (in the absence of MPH), without altering locomotion. In this way, a purely anxiolytic effect is observed compared to vehicle independent of motor function.

### U-Shaped Dose Effects of MPH on Aggressive Behavior

While most of the preclinical literature reports no effect and others show an anti-aggressive effect of MPH (24–26), our experimental settings enabled us to see the effects of MPH in a U-shaped manner. We observed an aggression-reducing effect of MPH when administered in a 3 mg/kg *i.p.* dose (i.e., the number of bites, attacks, and threats significantly reduced over time, and the attack latency got longer as well when compared to the vehicle and especially compared to the 10 mg/kg *i.p.* dose). On the first injection day, no clear differences were observed between vehicle and the 3 mg/kg *i.p.* dose. However, while aggression increased over time for the vehicle group, the level of aggression for the 3 mg/kg *i.p.* MPH-treated group remained more similar between testing days with regard to bites and attacks and even reduced for the number of observed threats, indicating a long-lasting effect of MPH. The 10 mg/kg *i.p.* MPH dose directly increased aggression on the first day, and this level remained the same on the last day, which was comparable to the level of aggression seen in the vehicle-treated group. Therefore, it can be argued that the 10 mg/kg *i.p.* dose increases aggression during the first resident–intruder interaction and appears to have no additional effect on aggression on the fifth day. While the 3 mg/kg *i.p.* dose does not have a direct effect on day 1, it is associated with a prolonged effect on attack latencies later on. The average attack latency of the last three interactions was significantly longer for the animals treated with 3 mg/kg MPH in comparison to both vehicle-treated and 10 mg/kg *i.p.* MPH-treated animals. Moreover, the number of attacks and threats were reduced in the animals treated with 3 mg/kg *i.p.* MPH on the last interaction day. This decrease in aggression could be caused by a reduction in social interest (or an increase in social trust) that we found in the social interaction test in BALB/cJ mice treated with 3 mg/kg MPH. Whether this is related to the state of anxiety remains unclear and needs to be investigated in more detail. While an increase in aggressiveness is not often reported as a consequence of MPH administration in rodents, an increase of aggressive behavior has been reported in humans as one of the side effects of MPH when it is administrated above the therapeutic window, probably as a result of inducing excessive increases in dopamine (DA) and noradrenaline (NA) levels (15, 45). Excessive DA and NA tone could bind to a larger population of DA and NA receptors, could increase the burden on monoamine reuptake transporters, and may even have spillover effects *via* volume transmission. Use of MPH outside the therapeutic window, due to therapeutic errors, accidental misdosage, misuse, or abuse may cause adverse effects that include irritability and agitation in a dose-dependent manner (46, 47). However, these adverse effects may be context dependent (48). While these effects are often observed in the clinic, they are rarely described in the literature (49). Therefore, we may assume the 10 mg/kg dose, which was given to BALB/cJ mice in our study, is outside the therapeutic window.

The U-shaped dose–response curve on aggression that we observed after administration of MPH may be related to differential effects on both DA and NA. In humans, MPH has a 50-fold lower effective dose for the noradrenaline transporter than for the DA transporter (50). A low dose of MPH (2.0 mg/kg *p.o.*; 0.25–1.0 mg/kg *i.p.*) has been shown to increase prefrontal cortex-dependent cognitive functioning by increasing both NA and DA efflux, without increasing locomotion. However, outside the prefrontal complex (PFC), these small doses had minimal impact on NA and DA release (51). With regard to NA, it is thought that the level of NA release determines which type of adrenoreceptor is activated. Moderate levels are thought to bind to high-affinity alpha-2a adrenergic receptors, whereas higher levels could also engage the lower-affinity alpha-1 and beta-1 adrenergic receptors (52). Studies that tested atomoxetine, a non-stimulant drug and selective NA reuptake inhibitor, show decreased anxiety (effect size 1.^51^, ^53^) and decreased aggression (effect size 0.52–1.10, 54, 55) after treatment in children with ADHD and comorbid disorders (e.g., anxiety disorder and oppositional defiant disorder; 56, 57). NA reuptake inhibitors (such as atomoxetine) have been demonstrated to increase DA levels in the PFC but not in the striatum and nucleus accumbens (58), while MPH administration increased DA levels in all these areas (59, 60). This may indicate that the aggression- and anxiety-reducing effects of MPH may be associated with the increase of NA or DA release specifically in the PFC.

We observed that administration of 10 mg/kg *i.p.* MPH causes the opposite effect (increased aggression), and excessive levels of DA and NA, caused by this higher dose of MPH, could have potentially driven the animal to a hyperarousal state (20, 52, 61). In addition, the high dose may also have activated other lower-affinity targets (e.g. the serotonin transporter; 62) that may have non-specific effects on the behavioral outcome by altering non-noradrenergic/non-dopaminergic mechanisms. It may be that MPH improves anxiety symptoms *via* the improvement of other ADHD symptoms such as attention (63), which together impact aggression by changing social attention. While an inverted U-shaped response of stimulant treatment has been demonstrated on attentional and working memory measures in both animals (64, 65) and humans (66), no preclinical study has shown a U-shaped dose effect of MPH on aggression before. Dissecting out the relative contribution of DA and NA receptor subtypes to the behavioral profiles of the 3 and 10 mg/kg *i.p*. MPH dose in BALB/cJ mice may be a useful next step.

## Conclusions

This study has investigated the effect of MPH on anxiety, fear extinction, and social behaviors including aggression in the BALB/cJ mouse. Independent of the dose, MPH reduced anxiety, whereas its effect on conditioned fear (decreased) was dose dependent. In addition, we found a differential effect of MPH on aggression in a dose-dependent manner. Specifically, administration of 3 mg/kg MPH *i.p.* prevented aggression escalation over time, while the 10 mg/kg *i.p.* dose increased the levels of aggression. This research may contribute to a better understanding of the efficacy of MPH administration and assist in understanding its potential impact in the clinical management of conduct problems and callous unemotional traits.

## Data Availability Statement

The datasets generated for this study are available on request to the corresponding author.

## Ethics Statement

The animal study was reviewed and approved by the Committee for Animal Experiments of the Radboud University Medical Center, Nijmegen, The Netherlands under project number: DEC2013-235.

## Author Contributions

AJ and JG were involved in the study conception. AJ, DK and FG performed the experiments. AJ, DK, FG, JB, TK and JG were involved in the data analysis and interpretation. AJ, JB, TK and JG were involved in the preparation and finalisation of the manuscript for publication.

## Funding

The research leading to these results has received funding from the European Community’s Seventh Framework Programme (FP7/2007–2013) under grant agreement nos. 603016 (MATRICS), 278948 (TACTICS), and 602805 (Aggressotype) and Horizon 2020 Research and Innovation Programme under grant no. 728018 (Eat2beNice).

## Conflict of Interest

The authors declare that the research was conducted in the absence of any commercial or financial relationships that could be construed as a potential conflict of interest.

The handling editor declared a shared affiliation, though no other collaboration, with the authors.
