# Supplementary material for: Methylphenidate Dose-Dependently Affects Aggression and Improves Fear Extinction and Anxiety in BALB/cJ Mice
Source: Front Psychiatry. 2019 Oct 25;10:768. doi: 10.3389/fpsyt.2019.00768 (PMC6823535; doi:10.3389/fpsyt.2019.00768)
Supplement: Supplementary file 1 [file DataSheet_1.docx]

Supplementary figures


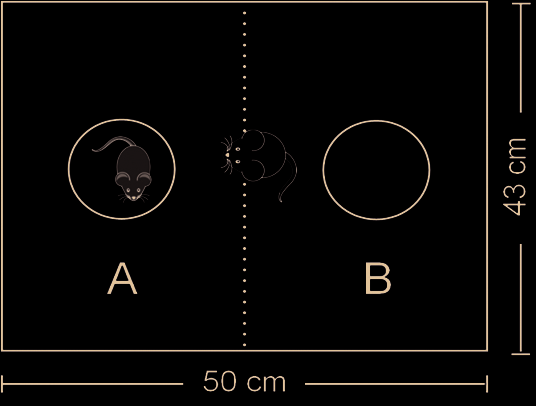


**Figure S1. Social interaction test.** Representation of testing arena. Two wire-mesh cylinders with large open ventilation holes (lxwxh 10x10x11 cm) were placed upside-down in a clear observation cage (dimensions 43x50cm) with corn bedding material on the floor. An unfamiliar C57BL/6J mouse was placed under a randomly assigned cylinder. Subsequently, a BALB/cJ mouse was placed in the middle of the cage and behaviour was recorded for five minutes using a high-speed infrared camera.


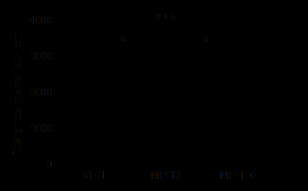

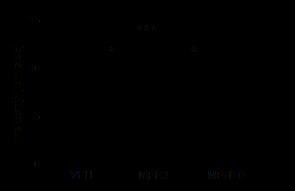


**Figure S2. Elevated plus maze** (a) distance travelled was dose-dependently increased after MPH administration. (b) Dose-dependent increase in velocity was observed after MPH administration. Abbreviations: VEH, vehicle; MPH3, 3 mg/kg i.p. methylphenidate; MPH10, 10 mg/kg i.p. methylphenidate. N=12 per group, * p < 0.05, *** p < 0.001.


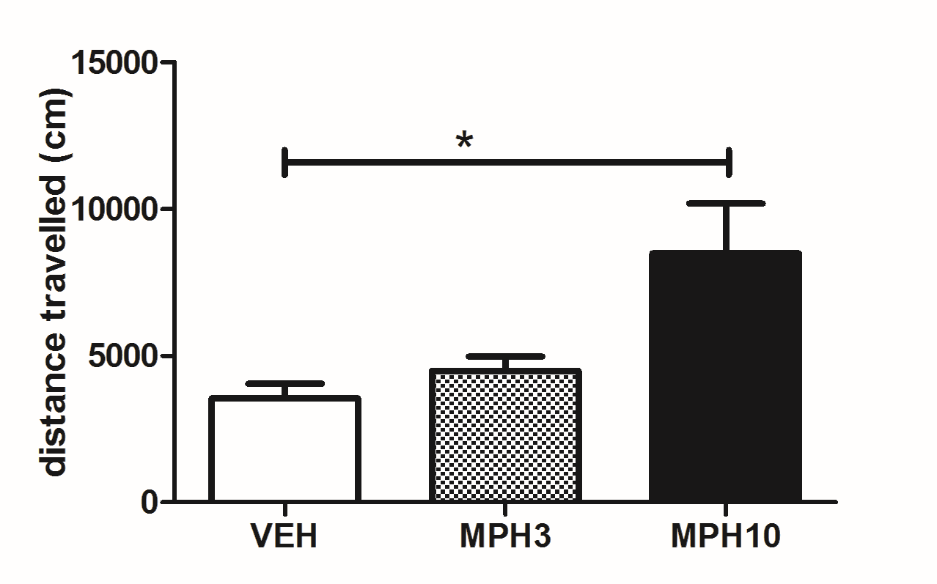


**Figure S3. Circular corridor** **test**. Distance travelled in the first 15 minutes in the circular corridor test. Increased locomotor activity was observed in the group treated with 10 mg/kg i.p. MPH. Abbreviations: VEH, vehicle; MPH3, 3 mg/kg i.p. methylphenidate; MPH10, 10 mg/kg i.p. methylphenidate. N=4 per group, * p < 0.05.


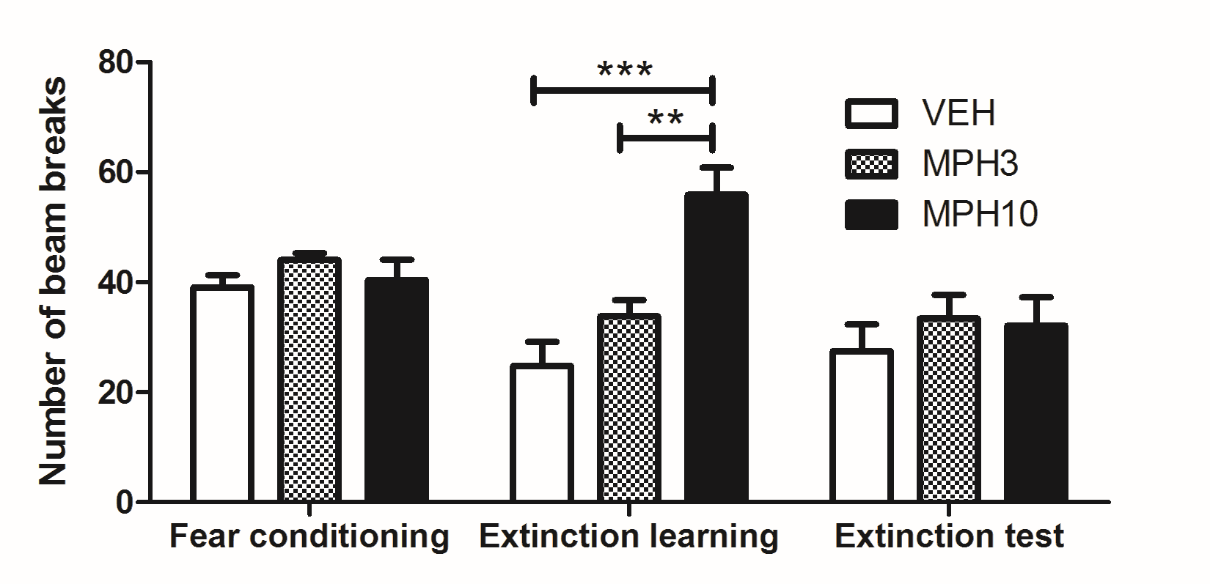


**Figure S4. Mixed cue fear conditioning** **test**. The number of beam breaks are presented during the habituation phases of the fear conditioning, extinction learning and extinction test. Abbreviations: VEH, vehicle; MPH3, 3 mg/kg i.p. methylphenidate; MPH10, 10 mg/kg i.p. methylphenidate. N=12 per group, ** p < 0.01; *** p < 0.001.
